# Feedback activation of neurofibromin terminates growth factor-induced Ras activation

**DOI:** 10.1186/s12964-016-0128-z

**Published:** 2016-02-09

**Authors:** Anne Hennig, Robby Markwart, Katharina Wolff, Katja Schubert, Yan Cui, Ian A. Prior, Manuel A. Esparza-Franco, Graham Ladds, Ignacio Rubio

**Affiliations:** Institute of Molecular Cell Biology, Center for Molecular Biomedicine, University Hospital, Hans-Knöll-Str.2, 07745 Jena, Germany; Leibniz Institute for Age Research - Fritz Lipmann Institute, 07745 Jena, Germany; Division of Cellular and Molecular Physiology, Institute of Translational Medicine, University of Liverpool, Liverpool, L69 3BX UK; Systems Biology Doctoral Training Centre, University of Warwick, Coventry, CV4 7AL UK; Department of Pharmacology, University of Cambridge, Cambridge, CB2 1PD UK; Center for Sepsis Control and Care, University Hospital, 07747 Jena, Germany

**Keywords:** GAP, GEF, Neurofibromin, NF1, Ras, Rsk

## Abstract

**Background:**

Growth factors induce a characteristically short-lived Ras activation in cells emerging from quiescence. Extensive work has shown that transient as opposed to sustained Ras activation is critical for the induction of mitogenic programs. Mitogen-induced accumulation of active Ras-GTP results from increased nucleotide exchange driven by the nucleotide exchange factor Sos. In contrast, the mechanism accounting for signal termination and prompt restoration of basal Ras-GTP levels is unclear, but has been inferred to involve feedback inhibition of Sos. Remarkably, how GTP-hydrolase activating proteins (GAPs) participate in controlling the rise and fall of Ras-GTP levels is unknown.

**Results:**

Monitoring nucleotide exchange of Ras in permeabilized cells we find, unexpectedly, that the decline of growth factor-induced Ras-GTP levels proceeds in the presence of unabated high nucleotide exchange, pointing to GAP activation as a major mechanism of signal termination. Experiments with non-hydrolysable GTP analogues and mathematical modeling confirmed and rationalized the presence of high GAP activity as Ras-GTP levels decline in a background of high nucleotide exchange. Using pharmacological and genetic approaches we document a raised activity of the neurofibromatosis type I tumor suppressor Ras-GAP neurofibromin and an involvement of Rsk1 and Rsk2 in the down-regulation of Ras-GTP levels.

**Conclusions:**

Our findings show that, in addition to feedback inhibition of Sos, feedback stimulation of the RasGAP neurofibromin enforces termination of the Ras signal in the context of growth-factor signaling. These findings ascribe a precise role to neurofibromin in growth factor-dependent control of Ras activity and illustrate how, by engaging Ras-GAP activity, mitogen-challenged cells play safe to ensure a timely termination of the Ras signal irrespectively of the reigning rate of nucleotide exchange.

**Electronic supplementary material:**

The online version of this article (doi:10.1186/s12964-016-0128-z) contains supplementary material, which is available to authorized users.

## Plain English summary

Ras activation in response to growth factor stimulation is a central mitogenic signaling pathway. Extensive work has shown that the duration of the Ras signal is a key determinant of cell fate in the sense that growth factor activation of Ras must be transient to promote a proper proliferative response. It is well established that growth factors stimulate the nucleotide exchange factor (GEF) Sos to promote Ras activation via Ras-GTP loading but it is not known how Ras activation is terminated to ensure a short-lived signal. We document here a new mechanism for Ras signal termination, namely activation of a RasGAP activity in the context of a feedback signal propagated via Rsk1 and Rsk2, the kinase mutated in Coffin-Lowry syndrome. We provide evidence that neurofibromin, the product of the tumor suppressor of neurofibromatosis type 1 (NF1), is the RasGAP species mediating the deactivation of Ras. In summary our findings disclose a positive feedback loop leading to the stimulation of neurofibromin as a mechanism that restricts the duration of growth factor-induced Ras activation.

## Background

Cells emerging from quiescence upon growth factor encounter feature a pronounced activation of Ras which is characteristically short-lived. Extensive work has elucidated that the duration of the signal elicited by Ras is decisive for cell fate decision taking. For example, seminal studies in PC12 phaechromocytoma cells illustrate that the duration of the signal provided by Ras and its downstream effector kinase Erk is the key event determining whether these cells will enter the cell cycle or cease proliferation and differentiate in response to a given stimulus [1–6]. Accordingly, the mechanisms mediating agonist control of Ras-GDP/GTP levels have been the focus of intense research.

Accumulation of active Ras-GTP in response to growth factors is understood in some detail. It results from the stimulation of the ubiquitous guanine nucleotide exchange factor (GEF) Sos and the consequent promotion of nucleotide exchange on Ras [7–10]. Less is known, however, about the reactions accounting for the equally fast reversal of Ras-GTP levels, a process we refer to as Ras *deactivation*. Current models invoke feedback inhibition of Sos as a critical step [11–14], based on the observation that Sos gets phosphorylated downstream of the Ras effector kinases MEK [15] and/or Erk [13, 16–18]. Erk phosphorylates multiple sites at the Sos C-terminus, promoting the dissociation of Sos from the adapter protein Grb-2 [16–19]. This reaction is inferred to down-modulate Sos activity by removing Sos from the vicinity of Ras, although not all studies are in support of this model [19–22].

Acting downstream of Erk, the two p90 ribosomal S6 kinase (Rsk) family members Rsk1 and Rsk2 have been identified as additional Sos kinases. Rsk2 phosphorylates Sos *in vitro* [23] and both Rsk1 and Rsk 2 reportedly phosphorylate Sos *in vivo* on two sites conforming to the minimal Rsk consensus motif [24]. Sos phosphorylation by Rsk creates docking sites for 14-3-3 proteins, and it is proposed that Sos/14-3-3 complex formation silences Sos activity [24]. Consistent with this model, preventing Sos phosphorylation by Rsk enhanced Erk activity but the effect was modest if compared to the consequences of MEK blockade, suggesting that modulation of Sos activity by Rsk1/2 is one out of many mechanisms accounting for the termination of Ras signaling. A role of Rsk1/2 in feedback control of Ras-GTP levels is further supported by studies illustrating that Rsk inhibition elevates the levels of activated Erk both at steady state or basal conditions [25–29] or in response to growth factor stimulation [24, 25, 30, 31]. Taken together these reports provide strong evidence for a feedback regulation of Ras-GTP levels mediated by Erk and/or Rsk1/2 impinging on Sos. However, it is worth to note that although Sos phosphorylation by Erk or Rsk1/2 is inferred to down-regulate Sos activity this link has not rigorously been proved since the nucleotide exchange activity of Sos was not analyzed in the referred studies.

While the role of Sos in Ras activation/deactivation has been intensively studied, the involvement of GTP-hydrolase activating proteins (GAPs) and in particular any mitogen-induced changes in GAP activity is less well explored. This lack of insight is owed not least to the fact that it is technically challenging to monitor GAP activity in life cells. Among the various human GAP families, neurofibromin, the product of the tumor suppressor gene NF1 has attracted particular attention given its frequent loss in human cancer [32, 33], which is strong circumstantial evidence for a function of neurofibromin in the control of mitogenic Ras signaling. As regards the precise role of neurofibromin, a recent series of studies has documented transient ubiquitination and proteasomal degradation of neurofibromin as a process contributing to the growth factor-induced accumulation of Ras-GTP [34–36]. The growth factor-triggered loss of neurofibromin protein was short-lived and related inversely with Ras-GTP levels, pointing to the short-term control of neurofibromin levels as one regulatory mechanism of Ras activation and deactivation. However, this mechanism may be restricted to certain cell types, since a growth-factor elicited drop of neurofibromin levels was not observed in other systems [37–40]. Thus, despite the strong interest in understanding neurofibromin function, the precise role played by neurofibromin in growth-factor control of Ras activity, if any, is still unclear.

In summary, the concept of feedback inhibition of Sos as the dominant mechanism of Ras deactivation has prevailed, perhaps due in part to the penury of data on Ras-GAP function in growth factor signaling. The need to advance in our understanding of Ras-GAP regulation is reinforced by mathematical simulations which predicted that Sos downregulation is insufficient to effectively deactivate Ras without invoking high GAP activity [41, 42]. Thus, while there is strong evidence pointing to a role for feedback inhibition of Sos in Ras deactivation, the full mechanism accounting for the transient nature of Ras activation is far from being understood. We have undertaken this study to elucidate the role of GEFs and GAPs during the process of Ras deactivation.

## Results and discussion

### EGF induces transient Ras activation and feedback phosphorylation of Sos

**Fig. 1:**
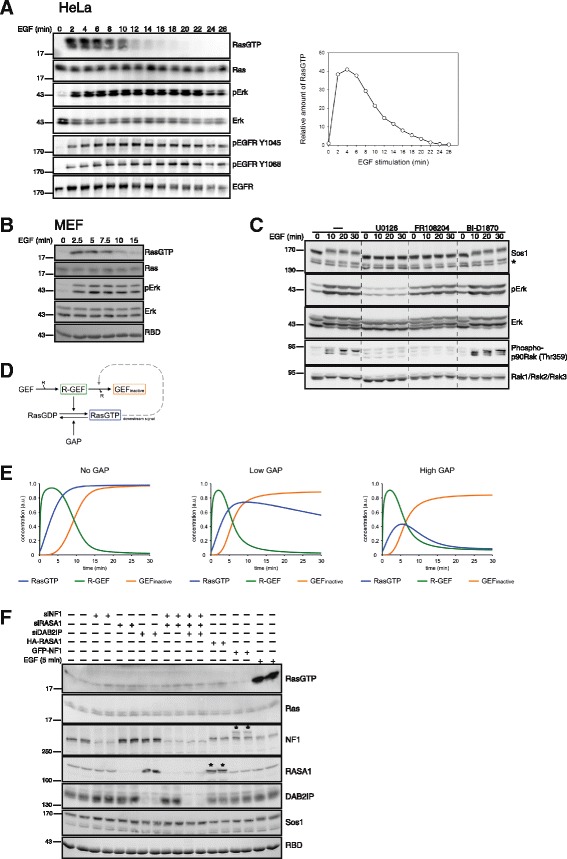
EGF induces transient Ras activation and Sos phosphorylation. **a** Transient Ras activation in HeLa cells. Serum-starved HeLa cells were challenged with 10 ng/ml EGF and Ras activation was determined via Ras-GTP affinity pulldowns. EGFR and Erk phosphorylation were determined using phosphosite-selective antibodies. A quantification of the Ras-GTP kinetics is shown on the right. RBD: Coomassie stain of Ras binding domain used for collecting Ras-GTP. **b** MEF cells challenged with EGF were processed for Ras and Erk activity assays as in (**a**). **c** EGF induces a mobility shift in Sos. HeLa cells were treated with inhibitors for MEK (10 μM U0126), Erk (50 μM FR108204) or Rsk (10 μM BI-D1870) prior to stimulation with EGF. Extracts were processed via western blotting using the indicated antibodies. Asterisk marks an unspecific doublet band. **d** Minimal Ras model describing Ras deactivation as induced by Ras-GTP-dependent feedback inhibition of Sos. R-GEF: receptor-GEF complex. See experimental section for details. **e** Simulations of Ras activation/deactivation using the model from (**d**) in a background of absent, low or high basal GAP activity. **f** Biochemical analysis of Ras-GTP levels following manipulation of Ras-GAP levels. The indicated Ras-GAP species were knocked down by siRNA (siNF1, siRASA1, siDAB2IP) or transiently overexpressed in HeLa cells (GFP-NF1: GFP-neurofibromin fusion construct; HA-RASA1: HA-tagged RASA1; asterisks mark overexpressed polypeptides). 5 min EGF stimulation is shown as positive control

Cells challenged with growth factors feature transient Ras activation as shown here for EGF-stimulated HeLa (Fig. 1a) and MEF cells (Fig. 1b). Extensive work has put forward feedback phosphorylation and concomitant down-regulation of Sos activity downstream of the Ras/Erk pathway as a step involved in signal termination [11–14, 16–18, 23, 43]. In HeLa cells EGF induced a shift in electrophoretic mobility of Sos (a commonly used surrogate marker of Sos phosphorylation [11–14, 16–18, 23, 43]) that was fully abrogated by MEK or ERK inhibition but was only partially affected by the Rsk inhibitor BI-D1870 [26] or PI3K inhibition (Fig. 1c, Additional file 1: Figure S1). This finding was consistent with a negative feedback loop impinging on Sos downstream of Erk and possibly downstream of Rsk to initiate Ras deactivation. Of note, in the present study we did not consider effects of Ras-GEFs other than Sos given the restricted neuronal and hematopoetic tissue distribution of the other two well-established Ras-GEF families, RasGRP and RasGRF. In line with the reported pattern of distribution, transcriptomic data sets confirmed the absence of all RasGRP and RasGRF family members from HeLa cells (GEO dataset ID GSE6783) [44].

### High GAP activity is implicit to models of transient Ras activation

While the role of Sos in Ras activation has been intensively studied, the involvement of Ras-GAPs is less well characterized. To understand the contribution of GAPs we generated a minimal mathematical model describing sequential growth factor-induced Sos activation, Ras-GTP formation and a Ras-GTP-initiated feedback loop of Sos-inhibition (Fig. 1d, Additional file 2: Table S1) and simulated Ras activation/deactivation in the background of absent, low or high basal GAP activity (Fig. 1e). In line with previous simulations [41], this analysis showed that models invoking feedback inhibition of Sos require the implicit assumption of high basal GAP activity in order to reproduce rapid Ras deactivation.

To investigate the role of Ras-GAPs we assessed first their expression pattern. A proteomic study detected RASA1, also known as p120GAP, and neurofibromin, the product of the neurofibromatosis type 1 (NF1) tumor suppressor in HeLa cells [45]. Transcriptome analysis also detected RASA1 and neurofibromin in HeLa cells and no appreciable levels of RASA2, RASA3, RASA4, RASAL1 or RASAL2 [44]. We confirmed the predominant expression of RASA1 and neurofibromin in HeLa cells (Fig. 1f) and found also robust expression of DAB2IP, a GAP described as tumor suppressor in prostate cancer [46] (Fig. 1f). To understand if Ras-GAPs were active in resting HeLa cells, we investigated the consequences of manipulating Ras-GAP levels. Remarkably, single or combined knockdown of RASA1, neurofibromin and DAB2IP resulted in a negligible increase in Ras-GTP levels as compared to EGF stimulation (Fig. 1f), suggesting that GAPs were in a dormant, inactive state in resting cells.

### Growth factor induced Ras-GTP accumulation is transient but the rise in Sos activity is sustained

**Fig. 2:**
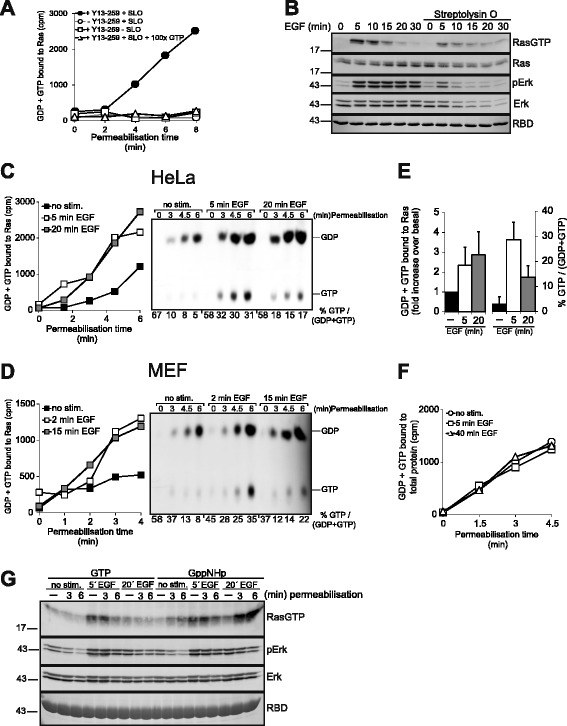
EGF induces transient Ras-GTP accumulation but sustained up-regulation of nucleotide exchange. **a** Specificity of the Ras nucleotide exchange assay in permeabilized cells. Serum-starved HeLa cells were permeabilized or mock permeabilized by omitting SLO in the presence of [α-^32^P]GTP. A 100fold molar excess of unlabeled GTP was included where indicated. Cell extracts prepared at the indicated time points were subjected to Ras-IPs or mock IPs lacking the Y13-259 Ras-antibody. Precipitates were washed and associated radioactivity evaluated by cerenkow counting. **b** Biochemical assay of time-dependent EGF-induced Ras and Erk activation performed in the absence or presence of the permeabilizing agent SLO. SLO was added simultaneously with EGF. **c** Nucleotide exchange assay in permeabilized HeLa cells before and 5 min or 20 min after EGF administration. Nucleotides associated to Ras-IPs were additionally eluted from Ras and separated via thin layer chromatography (TLC, on the right). %GTP/(GDP + GTP) values were determined by densitometry and plotted under the panel. Of note, initial values start off high and level off only at later time points. This pattern is owed to the different time required for single Ras proteins versus the whole Ras population to achieve steady-state nucleotide turnover. **d** Same experiment as in C performed in MEF cells. **e** Quantification of nucleotides bound to Ras-IPs. On the left, the amount of GDP + GTP bound to Ras at the 6 min assay point (as recorded in (**c**)) was plotted as the fold increase of radioactivity bound to Ras in EGF-stimulated versus unstimulated cells. On the right, the amount of GTP/(GDP + GTP) in the same assay points was plotted as % GTP/(GDP + GTP). Shown are means ± S.E.M. for three independent experiments. **f** [α-^32^P]GTP associated to total cellular protein from untreated or EGF-challenged permeabilized cells determined by a filter binding assay. Shown here is the means ± S.E.M. for three independent experiments. **g** GppNHp but not GTP promotes strong Ras activation in permeabilized cells at late time points of EGF stimulation. HeLa cells were permeabilized for the indicated time frames in the presence of GTP or GppNHp before (no stim.), 5 min or 20 min after EGF stimulation. Reactions were stopped by cell lysis and cell extracts were subjected to biochemical analysis of Ras and Erk activation

The absence of high Ras-GAP activity was difficult to reconcile with a model in which GAP action drives deactivation of Ras following feedback inhibition of Sos, unless Ras-GAPs became activated at later time points of growth factor stimulation. To understand the contribution of GAPs and GEFs we moved on to test these predictions experimentally. The common Ras-activation assay based on the affinity-precipitation of Ras-GTP does not inform on GEF/GAP activities because it measures steady-state levels of Ras-GTP which reflect the net result of GEF/GAP action. In order to monitor GEF and/or GAP activity we adapted an approach to assess nucleotide exchange on Ras at pre-steady-state in permeabilized cells [7, 8, 47]. As seen in Fig. 2a pulsing permeabilized HeLa cells with radiolabelled [α-^32^P]GTP leads to the time-dependent incorporation of radioactivity into Ras-immunoprecipitates (IPs). Radioactivity was specifically associated to Ras because it required the input of the permeabilizing agent Streptolysin O (SLO) and the Ras-IP antibody and was chased off by excess GTP (Fig. 2a). Importantly, permeabilization did not distort Ras activation kinetics although it did cause a moderate attenuation of the Ras-GTP amplitude and a progressive loss of Erk protein/activity beyond 10 min permeabilization time (Fig. 2b). It is important to note that all following permeabilization assays in this study involved maximum permeabilization times of 8 min or less.

To assess the role of Sos during rise and fall of Ras-GTP levels we monitored nucleotide uptake by Ras at discrete time points of EGF stimulation. EGF raised nucleotide uptake by Ras at 5 min stimulation (the peak of Ras-GTP accumulation) in HeLa (Fig. 2c) or MEF cells (Fig. 2d), consistent with the notion that EGF engages Sos to accelerate GTP-loading of Ras [7, 8, 47, 48]. Since Ras deactivation is predicted to involve feedback-inhibition of Sos we also measured nucleotide exchange at 15 or 20 min post-EGF, a time point at which Ras-GTP levels have reverted in MEF and HeLa cells, respectively (Fig. 1). Unexpectedly, we observed no reduction of nucleotide exchange at 15/20 min EGF in either cell line (Fig. 2c, d). To ascertain that Ras-GTP levels had truly declined under these experimental conditions, nucleotides associated to the same processed Ras-IPs were separated by thin layer chromatography (Fig. 2c, d). This analysis confirmed that Ras-GTP levels had strongly declined at 15/20 min EGF stimulation despite high nucleotide exchange in both cell types (Fig. 2e). Importantly, the association of [α-^32^P]GTP to total protein did not change with EGF stimulation (Fig. 2f), proving the specificity of the assay. While these data argued against a major drop in Sos activity during Ras deactivation, it should be noted that the permeabilization-based nucleotide exchange assay may not be sensitive enough to detect small and perhaps locally confined changes in GEF activity. Thus, although a contribution of Sos feedback inhibition could not be excluded on the basis of these findings, the simplest interpretation remained that Ras deactivation involved the stimulation of a GAP activity at late time points of EGF action.

To collect more evidence we combined the permeabilization assay with the Ras-GTP affinity pulldown. We reasoned that Ras-GTP loading driven by the uptake of the non-hydrolyzable GTP analogue GppNHp should accurately mirror Sos activity, because Ras-GppNHp is insensitive to GAP action. HeLa cells were permeabilized in the presence of GTP or GppNHp before or after EGF administration followed by the analysis of Ras activation (Fig. 2g). As expected, pulsing with GTP did not modify the pattern of Ras activation at any of the three stimulation time points chosen for permeabilization. Loading cells with GppNHp did not affect Ras-GTP levels before (null Sos activity) and 5 min after EGF stimulation (high Sos activity) as compared to GTP. However, GppNHp induced robust accumulation of active Ras at 20 min EGF, a time at which Ras-GTP levels have vanished in intact cells (Fig. 1a) or in permeabilized cells loaded with GTP (Fig. 2g). These observations indicated, firstly, that Sos is highly active at 20 min post-EGF, driving fast uptake of GppNHp by Ras, confirming the nucleotide exchange measurements shown in Fig. 2c and d. Secondly, the fact that Ras-GppNHp but not Ras-GTP accumulates at 20 min EGF proved the presence of high Ras-GAP activity during Ras deactivation. We concluded that Ras deactivation is enforced by an increase in GAP activity that counteracts high GEF activity at late time points of growth factor action.

### A negative feedback loop promotes Ras deactivation

**Fig. 3:**
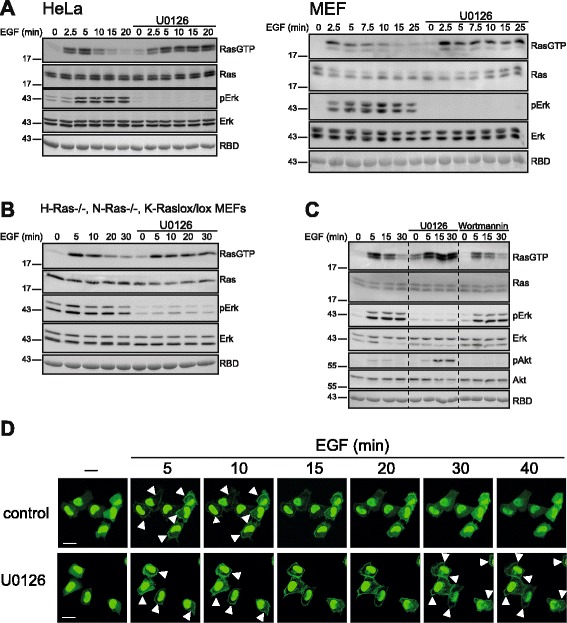
Inhibition of the MEK/Erk/Rsk pathway prolongs Ras activation. **a** Resting HeLa or MEF cells were left untreated or treated with the MEK inhibitor U0126 (10 μM), followed by EGF stimulation and analysis of Ras and Erk activity. **b** Same experiment as in A performed in H-Ras^-/-^, N-Ras^-/-^, K-Ras^lox/lox^ MEFs expressing only K-Ras. **c** HeLa cells pretreated with the MEK inhibitor U0126 or the PI3K inhibitor Wortmannin (30 min,100 nM) were challenged with EGF and subjected to a biochemical Ras activation assay. **d** HeLa cells expressing the trivalent affinity probe for Ras-GTP E3-R3(A/D) (see experimental section and Ref.[51]) were treated with U0126 or left untreated prior to stimulation with 10 ng/ml EGF. The time-dependent re-distribution of E3-R3(A/D) was imaged alive by confocal laser scanning microscopy. Probe relocation to the plasma membrane (marked by arrowheads) illustrates Ras activation. Over 30 cells monitored in 3-5 individual experiments responded with the same redistribution kinetics

Numerous studies have described a feedback loop acting via the Ras/Raf/MEK/Erk pathway through Sos inhibition to terminate Ras activation [13, 16–18, 43]. Most investigators postulated a role for Sos-phosphorylation in the inhibition of Sos activity and Ras deactivation but this aspect is disputed as others found that Ras activation kinetics were unaffected by the phosphorylation state of Sos [19, 20, 22]. Since we did not observe an inhibition of Sos during Ras deactivation we investigated the mode of action of the negative feedback. As reported previously [13, 15, 18], pharmacological inhibition of MEK using two distinct inhibitors or siRNA-mediated combined knockdown of MEK1/MEK2 prolonged Ras-GTP accumulation (Fig. 3a, Additional file 3: Figure S2). Operation of the feedback did not require cross-talk between the three Ras isoforms K-Ras, N-Ras and H-Ras because it proceeded in engineered MEFs expressing only K-Ras [49] (Fig. 3b). The feedback was specifically wired through the Erk pathway as it was not affected by inhibition of the Ras-effector PI3K (Fig. 3c). Finally, using trivalent fluorescent affinity probes for Ras-GTP [50, 51] to visualize Ras activation in life HeLa cells we ascertained that prolonged Ras activation following MEK inhibition reflected a uniform cellular response rather than a conglomerate of Ras activation kinetics (Fig. 3d).

### The feedback mechanism of Ras deactivation involves Erk and Rsk1/2 and stimulation of a RasGAP activity

**Fig. 4:**
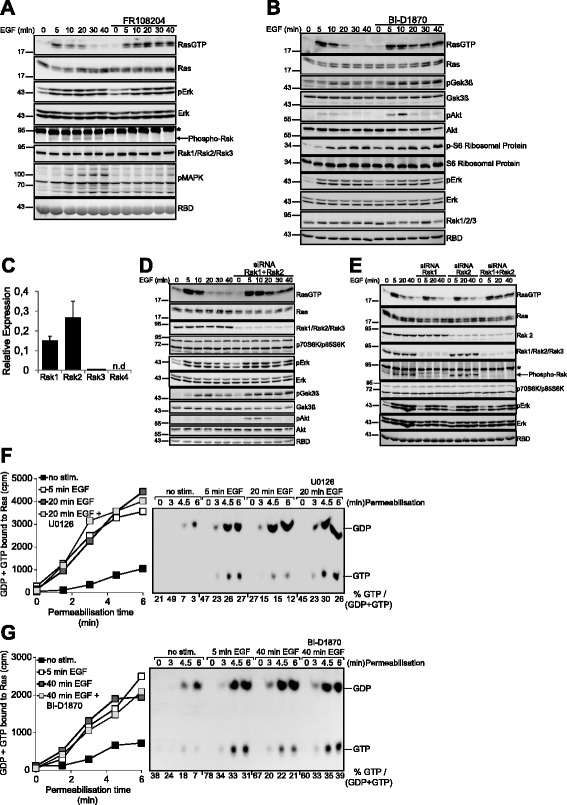
Rsk1 and Rsk2 mediate the feedback deactivation of Ras. **a** HeLa cells treated or not with the Erk inhibitor FR108204 were challenged with EGF for the indicated periods of time and subjected to a Ras-GTP pulldown assay. The phosphorylation/activation state of the indicated proteins was determined using phophosite-specific antibodies. Phospho-MAPK Substrates (PXS*P or S*PXR/K): Ab recognizing the phosphorylated Erk consensus motif. Asterisk denotes an unspecific band. **b** Same experiment in cells pre-treated with the pan-Rsk inhibitor BI-D1870. The activation status of Erk was monitored using phospho-site specific antibodies against Erk. Acute inhibition of Rsk with BI-D1870 did not affect Rsk protein stability as illustrated by the immunodetection of total Rsk1/Rsk2/Rsk3. **c** Real-time PCR analysis of Rsk isoform expression in HeLa cells. **d** Rsk1 and Rsk2 were simultaneously silenced via siRNA in HeLa cells followed by stimulation with EGF and biochemical analysis of Ras activation. **e** Biochemical determination of Ras-GTP levels and Erk activity in HeLa cells previously subjected to single or combined siRNA-mediated knockdown of Rsk1 and Rsk2. Immunodetection of p70S6K/p85S6K was performed as a control of specificity of the siRNA-mediated knockdown of Rsk1/2. Asterisk denotes an unspecific band. **f** Feedback deactivation of Ras is mediated via GAP up-regulation. HeLa cells were pretreated with U0126 where indicated, challenged with 10 ng/ml EGF and subjected to analysis of Ras nucleotide exchange. **g** Same experiment as in (**a**) performed in cells treated with the pan-Rsk inhibitor BI-D1870

To determine the signal path downstream of MEK, we inhibited Erk and Rsk, two downstream kinases that reportedly mediate feedback inhibition of the pathway [16, 17, 26, 29]. Inhibition of Erk (Fig. 4a) or Rsk (Fig. 4b) exerted a similar prolongation of Ras-GTP formation as MEK inhibition, albeit somewhat less potently in the case of Rsk. While assessing the specificity of Rsk inhibition, we noticed that phosphorylation of the Rsk substrate GSK3ß did not decline in cells treated with the Rsk inhibitor BI-D1870 (Fig. 4b). This was probably owed to the simultaneously proceeding activation of Akt (monitored by its phosphorylation on Ser473 in Fig. 4b), which was further enhanced in cells treated with the Rsk inhibitor. Akt phosphorylates the same residue on Gsk3ß as Rsk and both kinases have been shown to contribute to growth-factor-induced Gsk3ß phosphorylation in various cell types [26, 52]. To test more stringently the specificity of the Rsk inhibitor we also monitored phosphorylation of ribosomal protein S6, a target of p70-S6K, a kinase closely related to Rsk. Phosphorylation of ribosomal S6 protein was not affected by BI-D1807 treatment, supporting the specificity of the inhibitor. The involvement of Rsk in the negative feedback to Ras was further corroborated by combined RNAi-mediated knockdown of Rsk1 and Rsk2 (the two Rsk isoforms expressed in HeLa cells (Fig. 4c)), which produced a similar extension of Ras activation kinetics (Fig. 4d). Interestingly, the single knockdown of either Rsk1 or Rsk2 alone had no effect on Ras deactivation (Fig. 4e), indicating that the two kinase isoforms may perform redundant roles in feedback control of Ras activity. Along this line of thinking, the observation that the effect of Rsk inhibition on Ras inactivation (Fig. 4b and d) was less pronounced than the one caused by Erk blockade (Fig. 4a) suggested that Erk played a distinct role in the feedback deactivation of Ras independent from its role as upstream activator of Rsk. Interestingly, a similar collaboration between Erk and Rsk1/2 has been put forward before in the context of Sos feedback inhibition [24].

These findings evidenced that a negative feedback operating via Erk and Rsk1/2 mediates Ras deactivation. Since we did not observe an ostensible reduction in GEF activity during the decline of Ras-GTP levels (Fig. 2c, d) the feedback loop was unlikely to involve a strong down-regulation of Sos activity as the only mechanism of Ras deactivation. To test this hypothesis we investigated the consequences of interrupting the feedback on nucleotide exchange. Inhibition of MEK (Fig. 4f) or Rsk (Fig. 4g) restored Ras-GTP formation at 20 min or higher EGF in a background of unchanged strong nucleotide exchange. This finding provided further support for the concept that the feedback mechanism of Ras deactivation involves the activation of a Ras-GAP.

### Neurofibromin mediates Ras deactivation

**Fig. 5:**
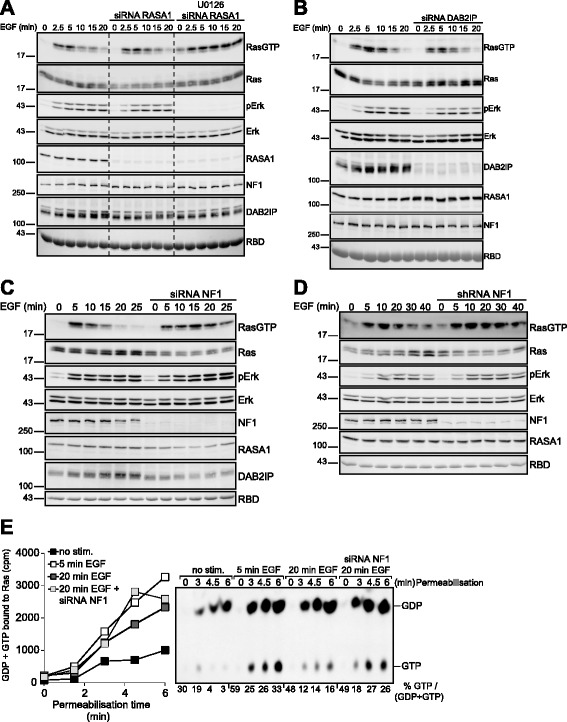
Feedback-mediated stimulation of neurofibromin mediates Ras deactivation. **a** EGF-induced Ras activation in HeLa cells subjected to previous siRNA-mediated silencing of RASA1. siRNA-transfected cells were additionally treated with the MEK inhibitor U0126 in order to ascertain that siRNA transfections did not distort the feedback mechanism of Ras deactivation. **b** Same experiment as in (**a**) in DAB2IP-silenced HeLa cells. **c** Same experiment as in (**b**) performed in neurofibromin-silenced HeLa cells. **d** Time course of EGF-driven Ras activation in HEK293T cells and a derivative line with stable shRNA-mediated knockdown of neurofibromin. **e** HeLa cells subjected to siRNA mediated silencing of neurofibromin were challenged with EGF. Cells were permeabilized prior to or 5 and 20 min after EGF stimulation and processed for the analysis of nucleotide exchange on Ras

In order to identify the Ras-GAP species involved in Ras deactivation we performed single knockdowns of RASA1 (Fig. 5a), DAB2IP (Fig. 5b) or neurofibromin (Fig. 5c). Remarkably, only knockdown of neurofibromin prolonged Ras-GTP accumulation. The same effect was observed in cells with stable shRNA-mediated knockdown of neurofibromin (Fig. 5d). Neurofibromin knockdown increased Ras-GTP accumulation 20 min post-EGF without affecting the rate of nucleotide uptake by Ras (Fig. 5e). This pattern was the same as obtained by pharmacological interruption of the feedback (Fig. 4a, b), corroborating that neurofibromin mediates the deactivation of Ras. Of note, we did not observe growth factor-dependent changes in neurofibromin levels in any of the cell types studied here (HeLa, HEK293 or MEF cells), which indicated that regulated neurofibromin ubiquitination/degradation as a mechanisms of Ras activity control does not occur in these cells [35, 36]. Taken together, our findings describe a precise role of neurofibromin in the control of Ras activity by growth factors and illustrate how, by engaging Ras-GAP activity, mitogen-challenged cells ensure a timely termination of the Ras signal irrespectively of the reigning rate of nucleotide exchange.

In 2004 Markevich et al. predicted on purely theoretical grounds that Sos downregulation was insufficient to effectively deactivate Ras [42] but many studies have continued invoking it as the basis of negative feedbacks in their models. Our finding that GEF activity does not decay significantly during Ras deactivation suggests that feedback inhibition of Sos is not the major mechanism of Ras deactivation, at least in the cell types studied here. Moreover, that model can only explain transient Ras activation by assuming high basal GAP activity (Fig. 1e), which was also not corroborated by our findings (Fig. 1f). Our data also provide evidence that feedback phosphorylation of Sos is not accompanied by a measurable decrease in nucleotide exchange and may therefore be more relevant for other signaling actions of Sos, perhaps related to the control of Rac and the actin cytoskeleton [53]. Interestingly, other investigators reached the same conclusion before by other means [54].

**Fig. 6:**
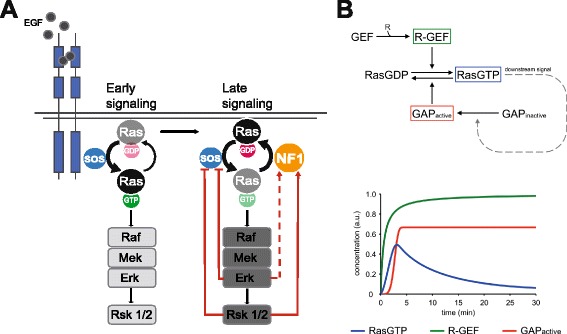
Model of Ras deactivation mediated by the feedback-dependent activation of neurofibromin. **a** Schematic cartoon of the mechanism of Ras activation/deactivation. The scheme depicts the previously reported Erk and/or Rsk-dependent feedback inhibition of GEF (Sos) activation and the feedback stimulation of neurofibromin reported herein. The dotted line linking Erk to neurofbromin symbolizes the presumptive Rsk-independent feedback loop emanating from Erk. See text for details. **b** Minimal mathematical model describing Ras activation/deactivation mediated by a positive feedback stimulation of Ras-GAP. R-GEF: receptor-GEF complex. See experimental section for details

Figure 6a and b show a mechanistic and a revised minimal mathematical model, respectively, that incorporate all present findings and can explain transient Ras activation by the sequential engagement of a GEF (Sos) and feedback stimulation of a GAP (neurofibromin) without the need to invoke other regulatory mechanisms. It is important to note that this model rationalizes all experimental findings reported herein for HeLa and MEF cells but alternative mechanisms involving other GEFs and/or GAPs may apply in other systems like neuronal or hematopoetic cells, which express a broader range of RasGEF and RasGAP species [55].

Extensive work suggests that the life-time of Ras activation has dramatic consequences on cell fate. In most investigated settings prolonged activation of the Ras/Erk pathway prohibits proliferation and shifts the balance to differentiation [1, 2, 56]. Our findings imply that loss of neurofibromin is likely to prolong rather than enhance the amplitude of Ras signaling, potentially providing a signal that is not compatible with excess proliferation, which could explain in part the absence of neoplastic growth in many tissues in neurofibromatosis. Interestingly, Schwann cells, the cell type most affected in neurofibromatosis, represent an exception to that rule, as enforced Ras/Erk signaling induces dedifferentiation of that cell type [57]. Our findings raise the possibility that aberrant prolongation of Ras-signaling owing to the loss of neurofibromin could drive dedifferentiation enabling aberrant growth in neurofibroma development.

Inactivating mutations in Rsk2 are causative of the Coffin-Lowry syndrome [58]. The involvement of both Rsk2 and neurofibromin in feedback deactivation of Ras suggests that both syndromes could share molecular mechanisms. In support of this notion, there are case reports of patients initially diagnosed with Noonan syndrome (one of several so-called rasopathies characterized by modest hyperactivation of the Ras/Erk pathway [59]) whose diagnosis was later on changed on the basis of re-sequencing to either NF1 or Coffin-Lowry [60]. Our finding of a negative feedback loop for Ras deactivation involving neurofibromin and Rsk2 rationalizes how inactivating mutations in Rsk2, a kinase with assumedly pro-mitogenic features, can give rise to a rasopathy-like phenotype.

## Conclusions

This study shows that transient Ras activation in response to growth factors is ensured by the sequential stimulation of Sos and the ensuing activation of the tumor suppressor Ras-GAP protein neurofibromin in the context of a feedback mechanism that involves also Rsk1/2. Hence, transient Ras signal termination does not result solely from feedback inhibition of Sos-driven nucleotide exchange but involves additionally feedback stimulation of neurofibromin RasGAP activity. This model of Ras activation represents a new paradigm and assigns for the first a time a precise role to neurofibromin in growth-factor-dependent control of Ras activity.

## Methods

### Cell culture and treatments/stimulations

Cervical cancer (HeLa) cells, Mouse embryonic fibroblasts (MEF), H-Ras^-/-^, N-Ras^-/-^, K-Ras^lox/lox^ MEFs (kindly provided by Mariano Barbacid, Madrid, Spain) and Human Embryonic Kidney 293 (HEK293T) cells were cultured at 37 °C and 5 % CO_2_ atmosphere in DMEM (Dulbecco’s modified Eagle’s medium) supplemented with 10 % (v/v) fetal calf serum. Inhibitor treatments: all inhibitors were applied for 30 min at the following concentrations: U0126 (10 μM), FR108204 (50 μM) BI-D1870 (10 μM), Wortmannin (100 nM). EGF was added at a final concentration of 10 ng/ml.

### Reagents

Streptolysin O was purchased from AaltoBio Reagents (Dublin, Ireland). Glutathione–Sepharose and all nucleotides were from JenaBioscience (Jena, Germany). [α-32P]GTP (370 MBq/ml; no. SCP-208) was from Hartmann Analytic (Braunschweig, Germany). GammaBind–Sepharose was purchased from Amersham Biosciences (Freiburg, Germany). U0126 and BI-D1870 were purchased from Enzo Life Science (Lörrach, Germany). The Erk1 and Erk2 selective inhibitor FR108204 [61] was from Sigma–Aldrich (Munich, Germany). All inhibitors were stored at -20 C in DMSO. Epithelial growth factor (EGF) were purchased from Life Technologies (Darmstadt, Germany). All siRNAs were ON-TARGETplus SMARTpools purchased from Dharmacon (Dharmacon RNAi Technologies, Thermo Fisher Scientific, Lafayette, USA): Human RASA1 (5921) (L-005276-00-0005), Human NF1 (L-003916-00-0005), Human DAB2IP (L-008249-01-0005), Human RPS6KA3 (L-003026-00-0005), Human RPS6KA1 (L-003025-00-0005), Map2k1 (L-003571-00-0005), Map2k2 (L-003573-00-0005). Saint-Red transfection reagent was from Synvolux Therapeutics (Groningen, Netherlands).

### Antibodies

Antibodies were obtained from the following sources: SOS1 (clone 25/SOS1), p120RasGAP (clone 13/RAS-GAP), MEK1 (no. 610121), MEK2 (no. 610235) were from BD Transduction Laboratories; K-Ras F234 (sc-30), N-Ras F155 (sc-31), pan-Ras C-4 (sc-166691), p-ERK1/2(Y204) (sc-101761), and Neurofibromin (sc-67) from Santa Cruz Biotechnology (Heidelberg, Germany); Phospho-MAPK/CDK Substrates (PXS*P or S*PXR/K) (34B2) (no. 2325), p44/42 MAPK (ERK1/2) (no. 4695), Akt (no. 9272), p-Akt(S473) (no. 4060), EGFR (no. 4267), p-EGFR (Y1068) (no. 2236), Phospho-p90RSK (Ser380) (no. 9341), RSK1/RSK2/RSK3 (32D7) (no. 9355), p-GSK-3β (Ser9) (D85E12) (no. 5558); GSK-3β (27C10) (no. 9315), Phospho-S6 Ribosomal Protein (Ser235/236) (no. 2211), S6 Ribosomal Protein (5G10) (no .2217), p70 S6 Kinase (49D7) (no. 2708) were from Cell Signaling Technology (Danvers, USA). Anti-DAB2IP (ab87811) was from Abcam (Cambridge, UK). Y13-259 rat monoclonal anti-Ras IP-antibody was purified from hybridoma supernatant (A.T.C.C., Manassas, U.S.A.).

### RNA isolation, cDNA synthesis and quantitative real-time PCR analysis

Total RNA isolation and purification was performed using an RNA isolation kit from Macherey-Nagel (Düren, Germany) according to the manufacturer’s protocol. cDNA synthesis was performed using First Strand cDNA Synthesis Kit (Thermo Scientific, Schwerte, Germany) with 50 ng/μl of total RNA per sample and Oligo-dT-based priming. QRT-PCR was performed using Maxima SYBR Green/ROX qPCR Master Mix (2X) (Thermo Scientific, Schwerte, Germany) using primers for all four RSK isoforms reported in [62]. Relative transcript levels were determined by calculating 2deltaCt values, using GAPDH expression levels for normalization.

### Plasmids and transient transfection

GFP-neurofibromin (type 1 isoform) was cloned in pCDH-EF1a-EGFP-C2-IRES-Puro, a customized vector based on the parental vector pCDH-EF1-EGFP-C2-IRES-Puro from System Biosciences, with expression driven by the EF1a promoter. Cloning details will be presented elsewhere. An expression construct for HA-tagged RASA1 [63] was kindly provided by Christian Widmann, University of Lausanne, Switzerland. Neurofibromin was stably knocked down in HEK293T cells via lentiviral transduction of a shRNA construct. The targeting sequence GCTGGCAGTTTCAAACGTAA embedded in a miRNA scaffold was cloned into pLV-H1-SGIPZ, a customized lentiviral vector based on pGIPZ vector (Open Biosystems). The resulting pLV-H1-SGIPZ-NF1sh1miR, together with psPAX2 (Addgene #12260) and pMD2.G (Addgene #12259), were transiently transfected into 293 T cells to produce lentiviral particles. 48 h post-transfection, the supernatant was harvested, filtrated through a 0.45 μM filter and used to infect 293 T cells. 48 h post-infection, puromycin selection was started to obtain the stable cell line. Transient transfections were performed using Polyethylenimine as described [64]. ON-TARGETplus siRNA-SMARTpool™ siRNAs were transfected using the Saint-Red transfection reagent from Synvolux Therapeutics exactly as described before [65].

### Ras-GTP pull-down assay

Cells seeded in 6 well plates were deprived of serum overnight, challenged or treated as appropriate and lyzed in 0.5 ml ice-cold lysis solution [50 mM Tris pH 7.5, 150 mM NaCl, 1 mM EGTA, 5 mM MgCl2, 1 % NP40 (Nonidet-P40)] supplemented with protease and phosphatase inhibitors, 100 μM GDP and 25 μg/ml soluble recombinant GST-RBD (Ras binding domain of Raf-1; previously produced in *E. coli* by standard procedures). GDP and GST-RBD were included in the lysis buffer to quench post-lytic GTP-loading and GAP-dependent Ras-bound GTP hydrolysis, respectively. Cell material was scraped off and lysates were cleared by centrifugation. GST-Raf-1-RBD/Ras-GTP complexes were collected on glutathione-sepharose (30 min at 4 °C on a rotating wheel), washed once with 750 μl lysis buffer lacking GDP and GST–Raf-1-RBD and processed for Western Blotting.

### Permeabilization and Nucleotide exchange assay

Cell permeabilization was performed essentially as described previously [66]. Serum-starved HeLa or MEF cells seeded in 6-well plates were treated/stimulated as appropriate and reactions were started by replacing the medium with 0.6 ml/well pre-warmed permeabilization solution (50 mM Hepes, pH 7.5, 107 mM potassium glutamate, 23 mM NaCl, 3 mM MgCl_2_, 0.1 mM CaCl_2_, 1 mM EGTA, 2 mM Dithiothreitol, 1 mM ATP) supplemented with freshly thawed 15 unit/ml SLO and 9 MBq [α-32P]GTP. For treated cells, this solution was supplemented with the relevant drug. Kinetics were started at this point and reactions were quenched by aspirating the solution and lysing cells in 1 ml/well ice-cold lysis buffer (50 mM Hepes, pH 7.5, 100 mM NaCl, 10 mM MgCl_2_, 1 % NP40, 100 μM GDP, 100 μM GTP and protease inhibitors) supplemented with 2,5 μg/ml Y13-259 Ras-antibody for IP. Cells were scraped off and extracts were placed on ice. Lysates were cleared by centrifugation and supernatants were made up to 500 mM NaCl, 0.5 %sodium deoxycholate and 0.05 % SDS. Immunocomplexes were collected on GammaBind–Sepharose by 45 min incubation at 4 °C under rotation. After six rounds of washing with 1 ml of ice-cold washing solution (50 mM Hepes, pH 7.5, 500 mM NaCl, 5 mM MgCl_2_, 0.1 % Triton X-100 and 0.005 % SDS), immunoprecipitates were subjected to Cerenkov counting. Ras nucleotides were eluted from the same samples and analyzed by Thin Layer Chromatography [66]. GDP and GTP spots were densitometrically quantified using Multi Gauge software.

### GEO search

The cervical carcinoma HeLa cells gene expression datasets reporting on the transcriptome of EGF-stimulated HeLa cells [44] were identified in GEO dataset ID GSE6783 with the platform ID GPL96.

### Confocal microscopy

Live-cell imaging was performed on a Zeiss LSM 510 axiovert confocal microscope equipped with a thermostated stage chamber (IBIDI, München, Germany) as previously described [67]. Briefly, confocal images (optical slice of ≤ 1 μm) were acquired using a 63x water immersion objective lens. EGFP was excited with the Argon 488 nm line and emitted fluorescence was collected with a 505–550 nm band-pass filter. All images of a series were exported as TIF files and subjected to the same processing routine using Zeiss ZEN 2008 Light Edition software.

### Mathematical modeling

A toy ODE model was built for two alternative network structures that represent regulation through GEF only (Fig. 1d), or GAP only (Fig. 6b). Both models share a common core to which we add a feedback loop that either decreases GEF activity or increases GAP activity. In the model Ras cycles between GDP and GTP bound states with Michaelis-Menten kinetics, the balance of which depends on the GEF/GAP ratio. To simulate EGF stimulation, receptor recruits GEF to form receptor-GEF complex (R-GEF) increasing the rate of Ras-GTP formation. Downstream signaling from Ras-GTP enforces the feedback after a small time delay to account for the MAPK cascade. In the GEF-only model, feedback catalyzes the separation of R-GEF complexes into free receptors and inactive GEF. In the GAP-only model, feedback catalyzes the activation of GAP molecules. The parameters of each model were adjusted to allow Ras-GTP dynamics to match Fig. 1a. All simulations were performed with the SimBiology toolbox within MATLAB R2013b. Details of the model are described in the supplementary information (Additional file 2: Table S1).

### Availability of supporting data

The data sets supporting the results of this article are included within the article and its additional file(s).

## Additional files

Additional file 1:
**EGF-induced electrophoretic mobility shift of Sos is sensitive to MEK (10 μM UO126) and Erk (50 μM FR180204) but not Rsk (10 μM BI-D1870) or PI3K (100nM Wortmannin) inhibition.** HeLa cells pre-treated with the indicated inhibitors or subjected to siRNA-mediated knockdown of neurofibromin (siNF1) were deprived of serum overnight and challenged 30 min with EGF. Lysates were processed for immunodetection of Sos, Rsk1/2/3 and Erk. (PDF 598 kb) Additional file 2:
**Details of the “GEF only” and “GAP only” model.** (PDF 174 kb) Additional file 3:
**Combined knockdown of MEK1 and MEK2 causes prolonged Ras activation. EGF-induced Ras activation was assessed biochemically after single or combined siRNA-mediated knockdown of MEK1 and MEK2.** RBD designates the coomassie-stained Ras binding domain used to collect Ras-GTP from cell lysates. Note that single knockdown of MEK1 or MEK2 leads to opposite effects on Ras-GTP levels, pointing to different roles of both kinases in Ras activity control. The same effect was previously reported by Kamioka et al. [13]. (PDF 28364 kb)

## Abbreviations

GAP: GTP-hydrolase activating protein
GEF: guanine nucleotide exchange factor
IP: immunoprecipitation
PM: plasma membrane
RBD: Ras binding domain
SLO: Streptolysin O
