# Supplementary figures and images for: Feedback activation of neurofibromin terminates growth factor-induced Ras activation
Source: Cell Commun Signal. 2016 Feb 9;14:5. doi: 10.1186/s12964-016-0128-z (PMC4746934; doi:10.1186/s12964-016-0128-z)

Figure S1

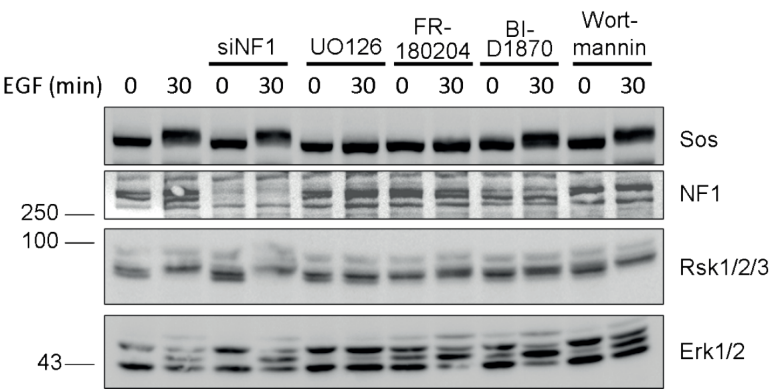

Supplement: Additional file 1: — EGF-induced electrophoretic mobility shift of Sos is sensitive to MEK (10 μM UO126) and Erk (50 μM FR180204) but not Rsk (10 μM BI-D1870) or PI3K (100nM Wortmannin) inhibition. HeLa cells pre-treated with the indicated inhibitors or subjected to siRNA-mediated knockdown of neurofibromin (siNF1) were deprived of serum overnight and challenged 30 min with EGF. Lysates were processed for immunodetection of Sos, Rsk1/2/3 and Erk. (PDF 598 kb) [file 12964_2016_128_MOESM1_ESM.pdf]

Figure S2

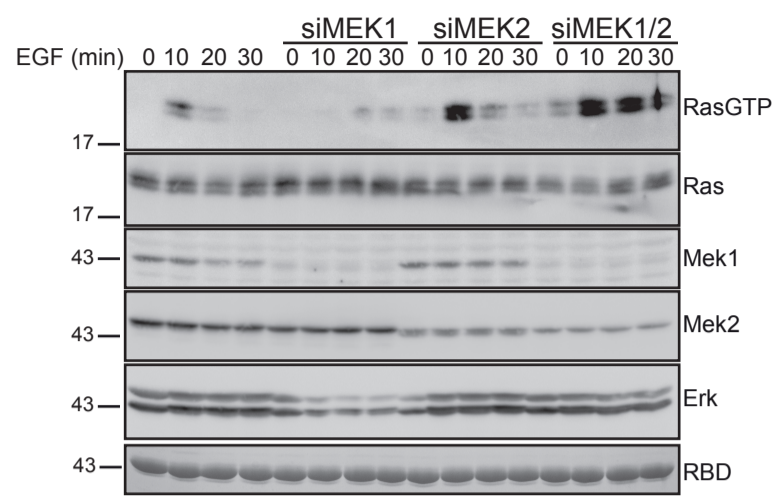

Supplement: Additional file 3: — Combined knockdown of MEK1 and MEK2 causes prolonged Ras activation. EGF-induced Ras activation was assessed biochemically after single or combined siRNA-mediated knockdown of MEK1 and MEK2. RBD designates the coomassie-stained Ras binding domain used to collect Ras-GTP from cell lysates. Note that single knockdown of MEK1 or MEK2 leads to opposite effects on Ras-GTP levels, pointing to different roles of both kinases in Ras activity control. The same effect was previously reported by Kamioka et al. [13]. (PDF 28364 kb) [file 12964_2016_128_MOESM3_ESM.pdf]
