# Supplementary material for: Feedback activation of neurofibromin terminates growth factor-induced Ras activation
Source: Cell Commun Signal. 2016 Feb 9;14:5. doi: 10.1186/s12964-016-0128-z (PMC4746934; doi:10.1186/s12964-016-0128-z)
Supplement: Additional file 2: — Details of the “GEF only” and “GAP only” model. (PDF 174 kb) [file 12964_2016_128_MOESM2_ESM.pdf]

Table S1

GEF-only model

ODEs:
d(RasGTP)/dt = ReactionFlux1 - ReactionFlux2
d(RasGDP)/dt = -ReactionFlux1 + ReactionFlux2
d(GEF)/dt = -ReactionFlux3
d([R-GEF])/dt = ReactionFlux3 - ReactionFlux8
d(R)/dt = -ReactionFlux3 + ReactionFlux8
d(delay1)/dt = ReactionFlux4 - ReactionFlux5
d(delay2)/dt = ReactionFlux5 - ReactionFlux6
d(delay3)/dt = ReactionFlux6 - ReactionFlux7
d([GEF-inactive])/dt = ReactionFlux8

Fluxes:
ReactionFlux1 = k1*[R-GEF]*RasGDP/(k2+RasGDP)
ReactionFlux2 =
k3*GAP_basal*RasGTP/(k4+RasGTP)
ReactionFlux3 = k5*R*GEF
ReactionFlux4 = kdelay*RasGTP
ReactionFlux5 = kdelay*delay1
ReactionFlux6 = kdelay*delay2
ReactionFlux7 = k6*delay3
ReactionFlux8 = k7*delay3*[R-GEF]

Parameter Values:
k1 = 0.005
k2 = 50
k3 = 0.05
k4 = 50
k5 = 0.001
k6 = 0.1
k7 = 1
kdelay = 0.001
GAP_basal = variable{Null=0, Low=1, High=8}

Initial Conditions:
RasGTP = 0
RasGDP = 100
GEF = 100
[R-GEF] = 0
R = 100
delay1 = 0
delay2 = 0
delay3 = 0
[GEF-inactive] = 0

GAP-only model

ODEs:

d(RasGTP)/dt = ReactionFlux1 - ReactionFlux2
d(RasGDP)/dt = -ReactionFlux1 + ReactionFlux2
d(GEF)/dt = -ReactionFlux3
d([R-GEF])/dt = ReactionFlux3
d(R)/dt = -ReactionFlux3
d(delay1)/dt = ReactionFlux4 - ReactionFlux5
d(delay2)/dt = ReactionFlux5 - ReactionFlux6
d(delay3)/dt = ReactionFlux6 - ReactionFlux7
d([GAP-active])/dt = ReactionFlux8
d([GAP-inactive])/dt = -ReactionFlux8

Fluxes:

ReactionFlux1 = k1*[R-GEF]*RasGDP/(k2+RasGDP)
ReactionFlux2 = k3*[GAP-active]*RasGTP/(k4+RasGTP)
ReactionFlux3 = k5*R*GEF
ReactionFlux4 = kdelay*RasGTP
ReactionFlux5 = kdelay*delay1
ReactionFlux6 = kdelay*delay2
ReactionFlux7 = k6*delay3
ReactionFlux8 = k7*delay3*[GAP-inactive]

Parameter Values:

k1 = 0.38
k2 = 10
k3 = 0.34
k4 = 0.01
k5 = 0.017
k6 = 0.01
k7 = 5.1
kdelay = 0.22

Initial Conditions:

RasGTP = 0
RasGDP = 100
GEF = 100
[R-GEF] = 0
R = 100
delay1 = 0
delay2 = 0
delay3 = 0
GAP_active = 0
GAP_inactive = 100
